# The Efficacy of Temperature-Controlled High-Energy Polymodal Laser Therapy in Tendinopathy of the Shoulder

**DOI:** 10.3390/jcm12072583

**Published:** 2023-03-29

**Authors:** Angela Notarnicola, Ilaria Covelli, Dario Macchiarola, Francesco Paolo Bianchi, Giuseppe Danilo Cassano, Biagio Moretti

**Affiliations:** 1 Orthopedics Section, Department of Translational Biomedicine and Neuroscience, Faculty of Medicine and Surgery, University of Study of Bari, General Hospital, 70121 Bari, Italy; 2 Course of Motor and Sports Sciences, Department of Precision and Regenerative Medicine and the Ionian Area, Faculty of Medicine and Surgery, University of Study of Bari, 70121 Bari, Italy; 3 Department of Interdisciplinary Medicine, Faculty of Medicine and Surgery, University of Study of Bari, General Hospital, 70121 Bari, Italy

**Keywords:** rotator cuff, tendinopathy, tendinitis, laser therapy, temperature-controlled high-energy laser therapy, HELT, wavelengths

## Abstract

Background: Rotator cuff tendinopathy is a common diagnosis among patients with shoulder pain and dysfunction. Laser therapy is recommended for the treatment of this tendon disease due to the possibility of increasing tissue biostimulation. The aim of this study was to investigate the effects of HELT (high-energy laser therapy) in relation to the wavelengths of 650 nm, 810 nm, 980 nm, and 1064 nm administered. Methods: The study design was prospective and observational. Thirty patients with shoulder tendinopathy were recruited and treated in one of two high-energy laser therapy groups (5 Watt/cm^2^, 450 Joule, super-pulsed mode). Group A received a high-energy laser therapy protocol with a single wavelength (1064 nm); group B received a high-energy laser therapy program with four wavelengths (650 nm, 810 nm, 980 nm, and 1064 nm). Pain (VAS), function (ASES), and disability (DASH) were monitored at the time of recruitment (T0), 1 month later (T1), and 6 months later (T2). Roles and Maudsley scores were also evaluated at T1 and T2. Results: Both protocols resulted in improvement of pain and in functional and disability recovery at the two times of assessment, without statistically significant differences. In group B, treated with the four wavelengths, a trend emerged, bordering on statistical significance, for a greater reduction in pain. Conclusions: The high-energy laser proved to be an effective therapy for the treatment of rotator cuff tendinopathy. The possibility of modulating the choice of wavelengths could allow the customization of the protocol in relation to the patient’s clinical condition.

## 1. Introduction

The cardinal features of rotator cuff tendinopathy are degenerative and inflammatory changes of the shoulder tendons. Rotator cuff tendinopathy is characterized by tendon-related pain with weakness—especially during elevation and external rotation—largely preserved range of motion, and minimal resting pain [1]. It accounts for more than half of all cases of chronic shoulder diseases [2]. The main purpose of rotator cuff tendinopathy treatment is to improve pain and physical disabilities. Treatment includes nonsteroidal anti-inflammatory drugs, analgesics, and corticosteroid injections. In addition, exercise and physical therapy have also been considered as nonpharmacological treatments.

Laser therapy, which stands for light amplification by stimulated emission of radiation, has been recommended to treat a wide variety of conditions, such as healing of wounds, bone repair, spinal cord injury, tendinopathy, postoperative recovery, and peripheral nerve regeneration [3]. In recent times, laser therapy—including low-power laser therapy and high-power laser therapy—has been used for the management of this disease [4]. Laser therapy is a non-invasive treatment with a low incidence of adverse effects. A meta-analysis showed that laser therapy is effective both alone and in association with other physiotherapy methods for shoulder tendinopathy [5]. In a randomized study [6], high-energy laser therapy (HELT), applied for pain treatment in tendinopathy, showed significant differences compared to low-energy laser therapy (LELT). HELT can stimulate the tendons more deeply and treat a wider area than LELT; thus, the application of HELT for tendinopathy may improve pain and function when compared to LELT [7]. However, studies regarding the effectiveness of HELT have been limited. Recently, high-energy laser devices have been able to modulate treatments, differentiating the choice of wavelength. Wavelengths of 650 nm, 810 nm, 980 nm, and 1064 nm are the most commonly administered. Each wavelength has different biological effects.

Thus, it is necessary to investigate the efficacy of different wavelengths of HELT in the treatment of rotator cuff tendinopathy. The aim of the present study was to perform a clinical trial to study the effects of HELT in relation to the wavelengths administered.

## 2. Materials and Methods

A prospective observational clinical trial was conducted. The study was approved by the local Ethics Committee (study number 6019, approved on 23 October 2019) and conducted according to the guidelines of the Declaration of Helsinki. Patients affected by rotator cuff tendinopathy, who came to our clinic, were enrolled in the study, and written informed consent was obtained from each patient. The following were used as inclusion criteria: aged between 25 and 90 years; capable of understanding; patients affected by rotator cuff tendinopathy characterized by pain in the proximal lateral part of the upper arm that is aggravated by abduction; a positive Jobe’s test and/or resisted external rotation test; a positive Hawkins–Kennedy test and/or Neer’s test; rotator cuff tendinopathy confirmed by ultrasonography of either tendon swelling, presence of hypoechoic areas, calcification, fibrillar disruption, and/or neovascularization in the supraspinatus muscle; symptoms for at least 3 weeks; indication of conservative treatment; pain in the previous week rated at last 5 on the pain scale (VAS 0–10); no neurological condition that could compromise muscle strength or activity; no history of shoulder surgery. The exclusion criteria were as follows: contraindications of laser therapy (e.g., tumors, the presence of a pacemaker or defibrillator, pregnancy); infiltrative and/or rehabilitative treatment in the two months prior to recruitment; pre-existing conditions associated with upper-extremity pain; difficulties in follow-up; depression; rheumatologic diseases; metabolic or systemic dysfunction.

The patients were treated with temperature controlled high-energy adjustable multimode-emission laser therapy (THEAL THERAPY, Mectronic Medicale, Italy). We used a super-pulse stochastic emission (E^2^C) characterized by random impulses emitted with a frequency varying between 20 Hz to 70 Hz, and a super-pulsed non-stochastic emission with a frequency in the range 15 Hz to 40 Hz. The device is equipped with a thermal control that is always active, with thresholds of 36–39 °C. Each patient was treated three times a week, for a total of six sessions. Each session lasted 5 min. The power was set constantly at 5 Watt, and in each session a total of 450 Joule was administered. The spot size was 1 cm^2^. The device enables the administration of the wavelengths as follows: 650 nm, 810 nm, 980 nm, and 1064 nm. Before starting the treatment, we tested the tolerance to the thermal pain threshold of the two protocols and assigned the patient to the one to which they showed greater tolerance. Patients in group A received a high-energy laser therapy with 1064 nm, thermocontrolled and adjusted. Patients in group B received a high-energy laser therapy, with a simultaneous adjustable combination of 650 nm (25%), 810 nm (25%), 980 nm (25%), and 1064 nm (25%), thermocontrolled and adjusted. All of the remaining energy parameters (Watts, minutes, Joules) were the same in both groups, as described above.

The patient was placed in a sitting position. The treatment surface area was the shoulder zone corresponding with the insertion of the rotator cuff on the humeral greater tuberosity. In a single session, each patient was treated with THEAL therapy using a dosage of 50 J per cm^2^ of the painful area at the level of the shoulder. The area was treated by carrying out the treatment following the direction from the rotator cuff muscle to the insertion on the humeral greater tuberosity, with a 90° inclination of the handpiece. As eye protection from the laser beam, all subjects wore protective darkened glasses. The patients were advised not to perform any other physical or pharmacological therapies during the study period. They were also advised to suspend work or sports activities that include repeated overhead movements or handling of loads greater than 15 kg.

For each patient, the epidemiological and anthropometric variables (i.e., age, gender, weight, height, Body mass index (BMI)) and the clinical characteristics (i.e., site of the disease, duration, clinical outcomes, and laser therapy protocol) were recorded. The primary endpoint was the response to treatment, defined as the reduction in pain on a VAS scale from recruitment to the follow-ups. The assessment times were at the time of recruitment (T0), at 1 month after recruitment (T1), and 6 months after recruitment (T2). The outcomes were the visual analog scale (VAS), the American Shoulder and Elbow Surgeons Standardized Shoulder Assessment Form (ASES), and the Disabilities of the Arm, Shoulder, and Hand (DASH) questionnaire. Possible adverse effects were monitored in both groups, including increased pain, joint lock, burn, and vagal crisis.

The present study aimed at testing the equality of the VAS scores in the two treatment groups: group A vs. group B. In order to test this hypothesis, the significance level (alpha) was set at 0.050, and a 2-tailed test was used. A sample size of 11 patients was proposed for each treatment group. With this sample size, and assuming a mean difference of 1.5 points in the VAS scores between groups (corresponding to the smallest effect that would be important to detect) and a common intragroup standard deviation of 1.3 (the hypothesis of the researchers, considering that we treated both groups with a high-energy laser, which in the literature has no precedents on the same pathological model), the study will have power of 82% to yield statistically significant results.

This analysis was carried out using Stata MP17 software. Continuous variables were expressed as the mean ± standard deviation and range, while categorical variables were expressed as proportions. The normality of the continuous variables was evaluated by means of the skewness and kurtosis test and, for those not normally distributed, a normalization model was set. Continuous variables were compared between groups using the Student’s t-test for independent data and between groups, times, and the interaction between them using ANOVA for repeated measures; a post hoc analysis was performed to estimate the variation of each outcome confronting each detection time per group. Categorical variables were compared between groups and times using the chi-squared test or Fisher’s exact test. Multivariate linear regression was used to evaluate the relationships in the differences between T2 and T0 of VAS, ASES, DASH, and various determinants; the correlation coefficient revealed a 95% confidence interval (95% CI). A *p*-value < 0.05 was considered statistically significant for all tests.

## 3. Results

The study sample consisted of 30 patients: 15 (50.0%) belonging to group A and 15 (50.0%) to group B. The epidemiological and clinical characteristics of the sample, by group, are described in Table 1. There were no significant differences between the two groups in relation to the variables analyzed. There were no dropouts or adverse effects.

In both groups, pain (monitored with VAS scores), function (quantified with the ASES scale), and disability (evaluated with DASH) showed a trend towards improvement at the two times T1 and T2 (Table 2).

Table 3 shows a statistically significant decrease in the VAS scores for both groups between T0 and T1 and between T0 and T2, and only for group B between T2 and T1 (*p* < 0.005). A statistically significant improvement in the ASES scores was observed for both groups between T0 and T1 and between T0 and T2, but only for group B between T2 and T1 (*p* < 0.005). The DASH scores decreased in both groups between T0 and T2 (*p* < 0.005); only group A significantly decreased between T0 and T1, while the variation was not significant between T1 and T2 for both groups.

The multivariate linear regression analysis revealed a statistically significant relationship of the difference between T2 and T0 of DASH and the onset of symptoms (coef. = 0.52; 95% CI = 0.08–0.95), showing that the treatment in the subjects with longer duration of symptoms assured a better decrease in DASH.

## 4. Discussion

To the best of our knowledge, there are no studies that have compared the effectiveness of different ways of delivering high-energy lasers. Therefore, in this study, the effects of two high-energy laser protocols that differed in the wavelengths administered were analyzed. The improvement proved to be significant in both follow-ups—namely, T1 and T6 (months)—with regard to painful symptoms (VAS), functional recovery (ASES), and disability (DASH), with no significant differences between the two protocols. On the other hand, there was a trend towards better values for pain remission in the protocol with the mix of four wavelengths (650 nm, 810 nm, 980 nm, and 1064 nm) compared to the one with only one wavelength (1064 nm). At the same time, there was a greater persistence of disability in patients who had a previous onset of symptoms. These data underline the need to start physical therapies as early as possible.

A laser is an electromagnetic radiation source characterized by monochromaticity, collimation, and coherence of the light beam; these characteristics prevent divergence and promote the propagation of energy into tissue. Laser therapy is a therapeutic option for physical therapy. The first therapeutic use of lasers occurred when the delivery of a 694 nm ruby laser caused the regrowth of shaved hair [8]. Photobiomodulation occurs through wavelength-dependent photon reception in the mitochondria; photons produce oxidation–reduction phenomena of cytochrome c oxidase, polarizing the mitochondrial membrane, activating adenosine triphosphate (ATP) synthetase [3], and inducing the dissociation of nitric oxide and production of reactive oxygen species [4]. This causes increases in metabolic turnover, vasodilation, angiogenesis, proliferation, and differentiation. The physiatrist sets a series of parameters (such as power, time, and wavelength) that determine the total emitted and absorbed photon dose [9]. Power defines the classes of lasers, with a higher power class (IV) producing > 0.5 W and the lower class (IIIb) producing 5 mW to 0.5 W. A class IV laser delivers a dose in less time than a class IIIb laser. Therapeutic lasers emit photons outside the visible spectrum, in the emission wavelength between 600 and 1070 nm, to meet the absorption spectrum range of cytochrome c oxidase. Wavelengths shorter than 600 nm are absorbed by hemoglobin and melanin, while wavelengths longer than 1070 nm are absorbed by water [4]. To determine the penetration into the tissue, the length of the laser must be in the near-infrared or visible-red spectrum (600 to 1070 nm). When the length is less than 600 nm, absorption by melanin and hemoglobin occurs, while when it is greater than 1070 nm, it occurs by water. Melanin also has an important absorption in the therapeutic window, with greater effects at lower lengths [9]. The laser transmission can be increased by higher powers (e.g., 3 W, 5 W), as well as by the delivery of multiple wavelengths together. In fact, the wavelength determines the depth of penetration, while the effect of the power is not yet clear. It is hypothesized that treatments with greater power (W) and shorter time allow the delivery of the total dosage necessary (J) more effectively, but with a greater risk of thermal damage [10].

Recent works have shown that both low-energy and high-energy lasers are effective on clinical–functional recovery in various tendon pathologies; in comparison, better results were found in favor of the high-energy laser [6] in relation to its greater ability to reach and stimulate deeper and wider areas [11]. A meta-analysis analyzed the clinical effects of different low-energy laser wavelengths in the treatment of elbow tendinopathy [12]; wavelengths of 820, 830, and 1064 nm produced negative results, while the wavelengths of 904 nm and 632 nm resulted in pain improvement and functional recovery, albeit in the short term. The peculiar aspect of this experience emerges in the differences between two high-energy laser protocols in the treatment of tendinopathy. Until now, no studies have compared the clinical effects of a high-energy laser in relation to the variability of wavelengths. Data suggest the possibility of modulating the treatment, differentiating the choice of certain wavelengths (650 nm, 810 nm, 980 nm, 1064 nm), which may offer therapeutic potential in rotator cuff tendinopathy. This clinical data are in agreement with the known specific biological effects of each wavelength administered. Furthermore, the combined administration takes advantage of the additional effects of each wavelength. The 650 nm length causes proliferation of mesenchymal cells, collagen, and fibronectin, and it avoids fibrosis by modulating the production of TGF-beta. The 810 nm length triggers the Hb oxidation process and promotes tissue regeneration. The 980 nm wavelength optimizes the action on thermal and mechanical receptors; it ensures correct interaction with the peripheral nervous system, activating the gate control mechanism for a rapid analgesic effect. The 1064 nm wavelength induces anti-inflammatory effects on connective tissue [13,14,15]. Furthermore, the super-pulsed E^2^C mode, which is characterized by a series of power impulses with variable duration and frequency, generates a rapid analgesic effect while limiting thermal effects on tissues.

This study has some important limitations, such as the short follow-up period and the low number of participants. Other limitations are that there was no control group, as well as the absence of blinding of the study. Moreover, there was no post-treatment ultrasound evaluation.

## 5. Conclusions

In conclusion, for the treatment of shoulder tendinopathy, the protocol with multiple wavelengths is a valid alternative to treatment with a single wavelength. These findings could be supported by further studies conducted using different doses and combinations of different wavelengths with more participants and longer follow-ups.

## Figures and Tables

**Table 1:** Characteristics of the sample, by treatment.

Variables	Group A (1064 nm)	Group B (650 + 810 + 980 + 1064 nm)	Total	*p*-Value
Age (years); mean ± SD (range)	60.7 ± 10.4 (45–75)	61.7 ± 13.5 (31–87)	61.2 ± 11.8 (31–87)	0.822
Females; *n* (%)	13 (86.7)	9 (60.0)	22 (73.3)	0.215
Weight (Kg); mean ± SD (range)	69.4 ± 13.0 (55–100)	71.1 ± 11.5 (51–90)	70.2 ± 12.1 (51–100)	0.713
Height (cm); mean ± SD (range)	163.6 ± 7.6 (150–180)	167.3 ± 9.9 (150–183)	165.5 ± 8.9 (150–183)	0.257
BMI; mean ± SD (range)	26.1 ± 4.5 (20.4–35.9)	25.1 ± 3.3 (19.2–30.0)	25.6 ± 3.9 (19.2–35.9)	0.495
Shoulder treated; *n* (%) Left Right	7 (46.7) 8 (53.3)	7 (46.7) 8 (53.3)	14 (46.7) 16 (53.3)	1.000
Beginning of symptoms (weeks); mean ± SD (range)	37.8 ± 15.2 (8–50)	34.4 ± 20.4 (8–62)	36.1 ± 17.8 (8–62)	0.412

**Table 2:** Mean ± SD and range of the VAS, ASES, and DSH variables by treatment and detection time, and repeated-measures ANOVA outcomes.

	T0	T1	T2	Comparison between Groups	Comparison between Times	Interaction between Times and Groups
VAS						
Group A	7.8 ± 1.5 (5–10)	5.6 ± 2.6 (1–9)	5.5 ± 3.0 (0–9)	0.055	<0.0001	0.238
Group B	6.9 ± 1.1 (5–9)	4.9 ± 1.9 (1–7)	3.3 ± 2.5 (0–7)			
Total	7.3 ± 1.4 (5–10)	5.2 ± 2.3 (1–9)	4.4 ± 2.9 (0–9)			
ASES						
Group A	30.9 ± 14.7 (13.3–54.9)	48.0 ± 23.1 (15.0–92.6)	52.2 ± 26.1 (15.0–98.3)	<0.0001	0.155	0.518
Group B	41.9 ± 16.9 (15.0–68.3)	52.5 ± 22.1 (15.0–88.3)	65.3 ± 24.3 (15.0–92.6)			
Total	36.4 ± 16.5 (13.3–68.3)	50.3 ± 22.3 (15.0–92.6)	58.8 ± 25.7 (15.0–98.3)			
DASH						
Group A	45.6 ± 15.1 (19.3–70.0)	34.8 ± 18.0 (5.8–70.0)	29.8 ± 20.1 (2.6–70.0)	0.001	0.452	0.870
Group B	39.1 ± 23.1 (9.2–70.0)	31.3 ± 19.7 (5.0–70.0)	26.5 ± 19.7 (2.0–70.0)			
Total	42.4 ± 19.5 (9.2–70.0)	33.1 ± 18.6 (5.0–70.0)	28.2 ± 19.3 (2.0–70.0)			

**Table 3:** Outcome variations between times, by group.

		Group A		Group B	
Outcome	Time	Contrast (95%CI)	*p*-Value	Contrast (95%CI)	*p*-Value
VAS	T1 vs. T0	−2.2 (−3.5–−0.9)	0.001	−2.0 (−3.3–−0.9)	0.002
	T2 vs. T0	−2.3 (−3.6–−1.1)	<0.0001	−3.5 (−4.8–−2.3)	<0.0001
	T2 vs. T1	−0.1 (−1.4–1.1)	0.832	−1.5 (−2.8–−0.3)	0.017
ASES	T1 vs. T0	17.1 (6.2–27.9)	0.003	10.6 (−0.2–21.5)	0.055
	T2 vs. T0	21.3 (10.5–32.2)	<0.0001	23.3 (12.5–34.2)	<0.0001
	T2 vs. T1	4.3 (−6.6–15.1)	0.436	12.7 (1.9–23.6)	0.023
DASH	T1 vs. T0	−10.8 (−20.5–−1.1)	0.030	−7.8 (−17.4–1.9)	0.114
	T2 vs. T0	−15.8 (−25.5–−6.1)	0.002	−12.6 (−22.3–−2.9)	0.012
	T2 vs. T1	−5.0 (−14.7–4.7)	0.307	−4.8 (−14.5–4.9)	0.328

## Data Availability

The data presented in this study are available upon request from the corresponding author. The data are not publicly available due to privacy concerns.
